# An Optical Filter-Less CMOS Image Sensor with Differential Spectral Response Pixels for Simultaneous UV-Selective and Visible Imaging †

**DOI:** 10.3390/s20010013

**Published:** 2019-12-18

**Authors:** Yhang Ricardo Sipauba Carvalho da Silva, Rihito Kuroda, Shigetoshi Sugawa

**Affiliations:** Graduate School of Engineering, Tohoku University, 6-6-11-811, Aza-Aoba, Aramaki, Aoba-ku, Sendai, Miyagi 980-8579, Japan; rihito.kuroda.e3@tohoku.ac.jp (R.K.); shigetoshi.sugawa.d4@tohoku.ac.jp (S.S.)

**Keywords:** UV, CMOS, image, sensor, ultraviolet, imaging, differential, spectral, response, silicon

## Abstract

This paper presents a complementary metal-oxide-semiconductor (CMOS) image sensor (CIS) capable of capturing UV-selective and visible light images simultaneously by a single exposure and without employing optical filters, suitable for applications that require simultaneous UV and visible light imaging, or UV imaging in variable light environment. The developed CIS is composed by high and low UV sensitivity pixel types, arranged alternately in a checker pattern. Both pixel types were designed to have matching sensitivities for non-UV light. The UV-selective image is captured by extracting the differential spectral response between adjacent pixels, while the visible light image is captured simultaneously by the low UV sensitivity pixels. Also, to achieve high conversion gain and wide dynamic range simultaneously, the lateral overflow integration capacitor (LOFIC) technology was introduced in both pixel types. The developed CIS has a pixel pitch of 5.6 µm and exhibits 172 µV/e^−^ conversion gain, 131 ke^−^ full well capacity (FWC), and 92.3 dB dynamic range. The spectral sensitivity ranges of the high and low UV sensitivity pixels are of 200–750 nm and 390–750 nm, respectively. The resulting sensitivity range after the differential spectral response extraction is of 200–480 nm. This paper presents details regarding the CIS pixels structures, doping profiles, device simulations, and the measurement results for photoelectric response and spectral sensitivity for both pixel types. Also, sample images of UV-selective and visible spectral imaging using the developed CIS are presented.

## 1. Introduction

The rise of applications for new technologies such as artificial intelligence (AI), machine vision, and Internet-of-Things (IoT) has led to an increase on image sensor performance demands, for better data acquisition and analysis [1,2,3,4,5]. Those applications are known to have different requirements than the conventional applications of imaging for viewing and entertainment. For instance, among the wavebands outside the visible light spectrum, the ultraviolet (UV) waveband (200–400 nm) has already been reported to contain useful information for data acquisition, such as: detection of harmful substances [6], measurement of ozone concentration in ozonated water [7], gas concentration measurement in semiconductor processes chambers [8], flame detection [9], and so on [10,11,12,13]. As research continues for the development of new applications, there is an increasing demand for image sensors with high UV sensitivity, high selectivity of sensitivity to the UV waveband, and an easy manufacturing process for low cost. Furthermore, a sensor capable of obtaining UV light and visible light images simultaneously and with a simple optical structure without external filters is desirable for UV sensing applications under background visible or near-infrared (NIR) light.

In this research, our objective is to develop a complementary metal-oxide-semiconductor (CMOS) image sensor (CIS) with a simple manufacturing process and capable of multiband imaging of the UV and visible light spectra in a single exposure, therefore outputting separately UV-selective and visible light images. This sensor targets applications that require UV imaging under variable background non-UV light, and applications requiring UV and visible imaging simultaneously. For example, in flame detection systems to identify the flame position and the background visible light, that can be analyzed to avoid false alarms and to detect the presence of personnel.

Currently available UV image sensors usually employ wide-gap semiconductors [14], or employ highly UV sensitive charge-coupled device (CCD) or CMOS image sensors [15,16,17,18,19,20] combined with bandpass filters [7,21], or with a constant illumination background [20], or with time-shared illumination by controlling the light sources operation timing [22]. However, those methods cannot capture simultaneously UV-selective and visible light images. Performing UV and visible light imaging simultaneously with a single image sensor has the advantages of allowing for UV detection with background recognition, to improve the UV sensing accuracy by selecting target area for analysis. Also, it allows a simple, small, and a low-cost setup, when compared with UV and visible imaging using multiple image sensors.

Several works have been reported to be useful in achieving multiband imaging with a single CIS, mainly to obtain RGB color information and for NIR imaging. The conventional method is to use color filter arrays, such as a Bayer pattern for obtaining color information or Fabry-Perrot filters in hyper-spectral sensors [23]. Other methods perform selective waveband detection in different depths, sometimes combined with optical filter arrays. For example, previous works achieved color RGB information for each pixel without demosaicing by stacking vertically B-, G-, and R-sensitive organic photoconductive films [24], or by using silicon (Si) with a triple-well structure to stack vertically three layers of pixels, therefore leveraging the relationship between light penetration depth with the wavelength in Si [25]. Another approach combines the depth sampling with filter arrays by stacking two CIS with color filters and reflectors, achieving high color fidelity in low light [26]. Also, a CIS has been reported to achieve RGB and NIR imaging simultaneously by using 3D stacking of two substrates, a color filter array on top substrate and an IR reduction algorithm [27].

Those approaches, however, are difficult to be extended for UV imaging, due to the following two reasons. First, it is difficult to obtain on-chip UV bandpass filters, necessary to selectively detect the UV waveband by filtering the incident light. Second, UV light has a very short penetration depth in silicon, with more than 90% of the incident light being absorbed in less than 30 nm from the surface. An approach trying to selectively detect the UV waveband by controlling the photodiode sensitivity range in the depth direction to the UV penetration depth would require charges to be detected only near the silicon surface. Extracting signal directly from such a thin surface layer leads to high dark current problems due to the interface states in-between the silicon surface and the silicon oxide layer. Hence, a new approach is necessary for UV light multispectral imaging.

**Figure 1:**
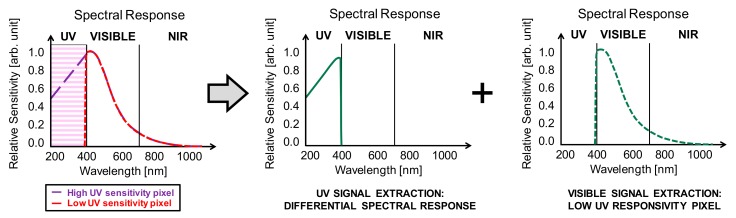
Differential spectral response method used in the developed CMOS image sensor (CIS) to obtain visible light and UV-selective light images simultaneously. This method uses high and low UV sensitivity pixels. The UV-selective image is obtained from the differential spectral response of both pixel types, and the visible light image is simultaneously obtained from the low UV sensitivity pixels.

In this work, UV light detection is carried out by leveraging the differential spectral response between pixels with high and low UV sensitivity, as explained in Figure 1. We have previously used this approach to develop a discrete silicon UV radiation sensor by monolithically integrating high and low UV sensitivity photodiodes [28,29]. In this report, the technology was further developed for pinned photodiode with full charge transfer capability, suitable for CIS implementation, and we report an optical filter-less CIS outputting UV-selective and visible light images simultaneously in a single exposure by employing the differential spectral response method. The developed technology does not require special processes when compared with conventional CIS, and can be achieved by adding only one single photomask and a few additional ion implantations to a conventional CIS. 

The structure of this paper is as follows. In Section 2, information about the sensor block diagram and the pixel structure considered when designing the CIS is presented, with detailed description of the simulation setup and simulation results for the internal potentials, donor concentration, acceptor concentration, charge transfer, and spectral sensitivity for each type of photodiode. Section 3 summarizes the chip manufacturing process and the measurement results, such as photoelectric transfer curve and spectral responses. Sample images captured with the manufactured sensor are also presented. Section 4 shows the conclusion of this work. This paper is the extended version of a previous report [30], with detailed design and simulation information and added measurement results.

## 2. Sensor Design, Fabrication, and Measurement Setup

In this section, we describe the block diagram of the external circuits used in the developed CIS, discuss the considered pixel structures and dopant concentration profiles, describe the simulations procedures, and show the simulation results performed for the CIS design. The simulations carried out were the charge transfer from inside the photodiode to the transfer gate, simulated by analyzing the potential inside the photodiode to check for potential pockets capable of trapping the charges during transfer operation, and spectral internal quantum efficiency (internal QE) in the waveband of 200–1000 nm.

### 2.1. Circuit Architecture

**Figure 2:**
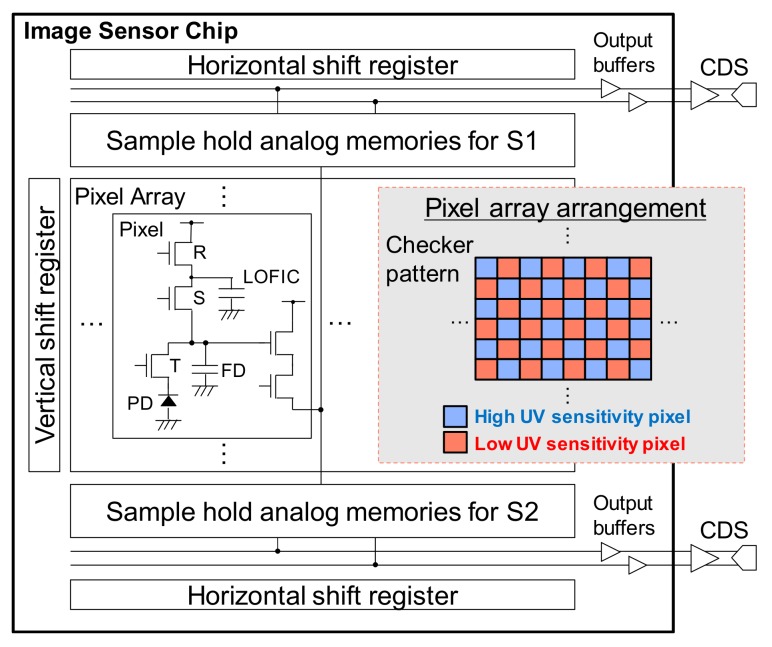
Circuit architecture and pixel array arrangement. Pixels were arranged in a checker pattern to facilitate the differential signal extraction. A lateral overflow integration capacitor (LOFIC) was implemented in each pixel.

Figure 2 summarizes the circuit block diagram of the developed CIS and the pixel array arrangement. The pixel structure is similar to a 4T pixel, but with an S switch and a lateral overflow integration capacitor (LOFIC) [31] added to achieve high dynamic range by performing charge-voltage conversion in the small floating diffusion (FD) capacitance in low light illumination, and in the large FD + LOFIC capacitance in strong light conditions. The developed sensor is composed by two types of pixel: pixels with high UV sensitivity (Pix. A) and low UV sensitivity (Pix. B), arranged alternately in a checker pattern as shown in Figure 2, in order to facilitate the differential spectral response extraction and interpolation. Optimization in the arrangement may be possible in future works. The sensor contains four analog memories for each column, two for the high conversion gain signal using FD (signal S1) and two for the high saturation signal using FD and LOFIC (signal S2). Each signal path has a horizontal shift register and two output buffers. Correlated double sampling (CDS) and digitizing operations are carried out off-chip. Details about the pixel’s structure are described in detail in Section 2.2.

### 2.2. Pixel Structure and Implant Profiles

**Figure 3:**
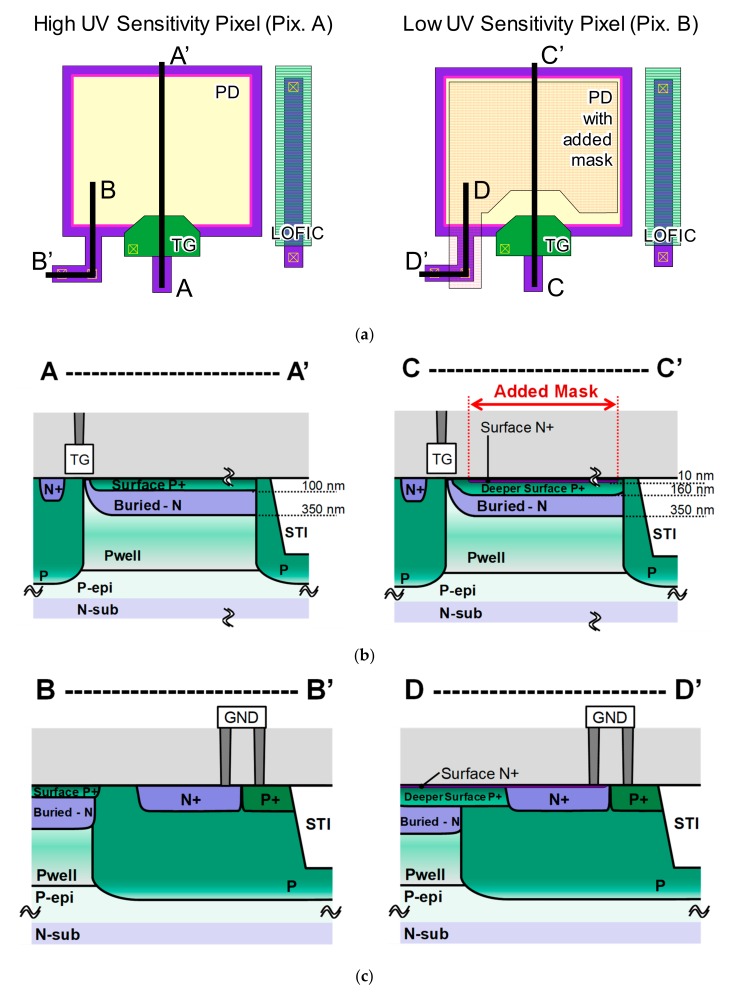
(**a**) The layout of both high UV sensitivity pixel (Pix. A) and low UV sensitivity pixel (Pix. B). (**b**) The cross-sectional structure diagram from inside the pixel to the transfer gate, and (**c**) the cross-sectional diagram alongside the pixel border.

Figure 3 shows details of the high UV sensitivity pixel and low UV sensitivity pixel structures. Figure 3a shows the aerial view of the pixel photodiode and LOFIC. Both pixel types have a similar design, differing only by the addition of one photomask in the low UV sensitivity pixel, shown in Figure 3a with a pink color. This photomask is used in three ion implantation processes: a surface N^+^, a deeper surface P^+^, and an additional buried-N implant. The objective of each implant added in Pix. B is as follows. For the surface N^+^ layer, it is to drain out electrons generated near the surface and, therefore, reduce UV light sensitivity. For the deeper surface P^+^ layer, it is to form a neutral p-type region between the surface N^+^ and the buried N layers, in order to prevent leakage current between both layers. Finally, for the additional buried-N layer, it is to compensate for the loss of N-type net doping concentration in the buried-N layer due to the added surface P^+^ implant, and achieve a similar potential profile to Pix. A in the charge transfer operation from the buried-N to the transfer gate and floating diffusion. In this sensor, a metal-oxide-semiconductor (MOS) capacitor structure was used for LOFIC.

In Figure 3, the transversal view alongside the lines A–A’ and C–C’ are shown in Figure 3b, and alongside B–B’ and D–D’ are shown in Figure 3c. As shown in Figure 3b, both photodiodes have a Pwell layer added to reduce sensitivity to visible and mainly to near-infrared (NIR) light, therefore improving the UV signal extraction accuracy by reducing detection of unwanted light wavebands even before the differential signal extraction. The Pwell implant in both pixel types used the same photomask as the buried N layer implantation. The high UV sensitivity pixel (Pix. A) has a thin surface P^+^ layer with a high concentration in its surface and a steep dopant profile, to form a drift electrical field from near the silicon surface to the buried N layer, therefore achieving high UV sensitivity [16,17]. In the low UV sensitivity pixel (Pix. B), a very thin and high concentration surface N^+^ layer was added to drain out or recombine electrons generated near the Si surface. It also features a deeper surface P^+^ layer to separate the surface N^+^ from the buried N layer, and an additional buried-N implant. Those implants were carried out by only one added photomask.

The potentials for the surface N^+^ in Pix. B and surface P^+^ in both pixel types are fixed and set to the ground level by an external electrode, as shown in Figure 3c. This is important to actively drain out electrons generated by UV light, that are generated near the silicon surface and are drifted to the surface N^+^ layer, instead of only relying on the carrier recombination to reduce UV light sensitivity. Applying a voltage in the surface N^+^ layer has the advantage of potentially allowing for direct UV detection form the surface N^+^ layer, without the need to extract the differential spectral response of Pix. A and Pix. B. However, this approach has several challenges that are avoided by the proposed differential spectral response method, such as: reverse leakage currents in the p-n junction with the surface P^+^ layer, spread of depletion width, leakage currents between the surface N^+^ and buried-N layers, and increased capacitance in the Pix. B surface.

Another important aspect of the low UV sensitivity Pix. B is the shape of the added photomask, shown in Figure 3a with a pink color. The photomask was added a few hundred nanometers away from the transfer gate, to separate the photodiode in two regions: a low UV sensitivity region, with the added implants, and a transfer region near the transfer gate, with a profile similar to Pix. A. This approach was used to allow for charge transfer from the photodiode to the FD region.

**Figure 4:**
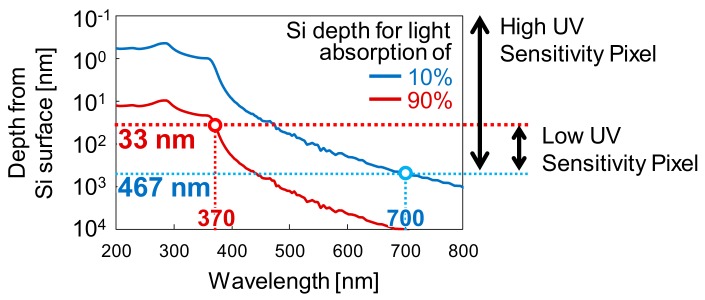
Calculated depth from the silicon surface where 10% and 90% of the incident light is absorbed, in function of the wavelength, and the target depths with light sensitivity for both high and low UV sensitivity pixel types.

Figure 4 shows the depth in Si where 10% and 90% of the incident light is absorbed, respectively in the blue and red lines, as a function of the wavelength. As shown in Figure 4, more than 90% of the UV light with wavelengths shorter than 370 nm is absorbed within the first 33 nm from the silicon surface, while less than 10% of NIR light is absorbed until a depth of 467 nm. Therefore, the target depths for the detection of the photo-generated charges, i.e., depths where each type of pixel photodiode should have sensitivity, are defined as follows. For Pix. A, from the Si surface (depth of 0 nm) to approximately 500 nm, in order to achieve high UV sensitivity while reducing NIR light sensitivity. For Pix. B, from a depth of approximately 33 nm from the Si surface to approximately 500 nm, in order to selectively reduce UV light sensitivity while matching the visible and NIR light sensitivity with Pix. A.

**Figure 5:**
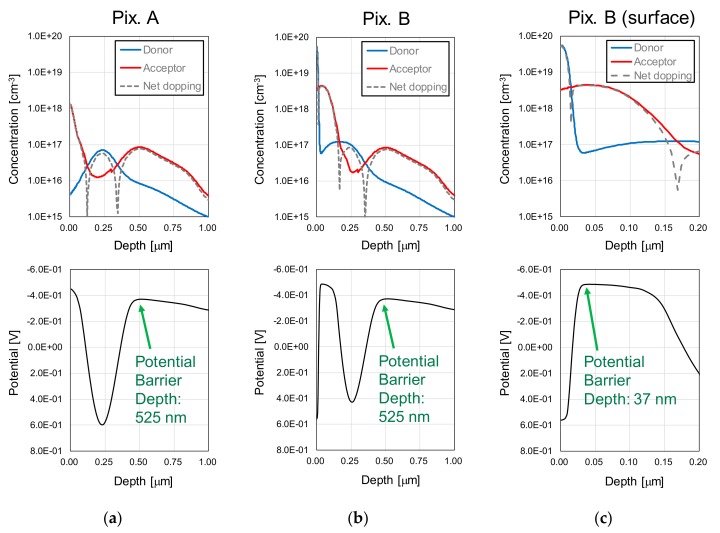
Implant concentration profiles and potential profiles in the depth direction for (**a**) the high UV sensitivity pixel (Pix. A), (**b**) the low UV sensitivity pixel (Pix. B), and (**c**) the surface region of the low UV sensitivity pixel.

Figure 5 shows the implant profiles and the respective calculated internal potential profiles for each type of pixel. Figure 5a shows for the high UV sensitivity pixel (Pix. A), Figure 5b for the low UV sensitivity pixel (Pix. B), and Figure 5c shows Pix. B profiles near the Si surface. From the implant profiles and the net doping concentrations, the p-n junction depths are as follows. For Pix. A, the junction formed between the surface P^+^ and the buried N layer is at 100 nm, and between the buried-N and the Pwell layers is at 350 nm, approximately. For Pix. B, the junction between the surface N^+^ and surface P^+^ layers is at 10 nm, the surface P^+^ and buried N layers at 160 nm, and the buried-N and Pwell layers at 350 nm, approximately.

The potential profiles show that, in both Pix. A and Pix. B, the p-n junction between the buried-N and the Pwell layers forms a potential barrier at a depth of 523 nm from the Si surface. This potential barrier depth is important to reduce visible and mainly NIR sensitivity, since charges generated at greater depths cannot reach the buried-N detection layer due to the potential barrier. In the surface region, Pix. A has a potential gradient formed from the Si surface to the buried-N, to achieve high UV sensitivity by drifting surface charges to the buried-N layer, while Pix. B has a potential barrier between the surfaces N^+^ and P^+^ layers at a depth of 37 nm, to selectively reduce UV sensitivity. Therefore, the internal profiles show that sensitivity ranges similar to the target values shown in Figure 4 are expected.

In this approach, since pixel sensitivity is modified only by controlling the photodiode internal potentials by ion implantations, it is possible to reduce sensitivity selectively to the UV waveband in Pix. B, in a ratio and with an accuracy determined by the relationship of the target waveband and its absorption coefficient in Si. Even after the differential spectral response (Pix. A–Pix. B), it is expected some residual sensitivity to visible light, mainly to the blue waveband, because non-UV light absorption also occurs within the first 37 nm from the surface. Decreasing the depth of potential barrier between the surface N^+^ and surface P^+^ layers would reduce the residual sensitivity. However, for 20 nm or less, this comes at a cost of also increasing UV light sensitivity in Pix. B, as shown in our previous work [29]. Therefore, a potential barrier in the depth range of 30 nm to 40 nm was targeted for the sample manufacturing.

Other possible approaches for UV detection are to use only high UV sensitivity pixels and a filter array containing high and low UV transmittance filters, or to use only high UV sensitivity pixels combined with in-pixel UV bandpass filters. However, those approaches have crucial challenges. In the former approach, the variances in light transmittance for the visible and NIR light spectra between both filter types introduces errors in the signal extraction. In the latter approach, it is difficult to obtain on-chip UV bandpass filters in a low-cost, easy to manufacture process, and to capture simultaneously ultraviolet and visible light images.

### 2.3. Device Simulation

The pixel structures shown in Figure 3 and the dopant concentration profiles shown in Figure 5 were reproduced in the 3D device simulator SPECTRA, from Link Research Corporation, to analyze the charge transfer and calculate the spectral internal QE for both pixel types. Charge transfer was analyzed by calculating the potential profile from the pixel photodiode (PD) to the floating diffusion (FD) when the transfer gate (TG) is turned on. This approach was used to analyze the presence of potential pockets that trap the charges inside the PD, and was crucially important when determining manufacturing parameters such as the distance from the added mask to the transfer gate and the doping conditions.

**Figure 6:**
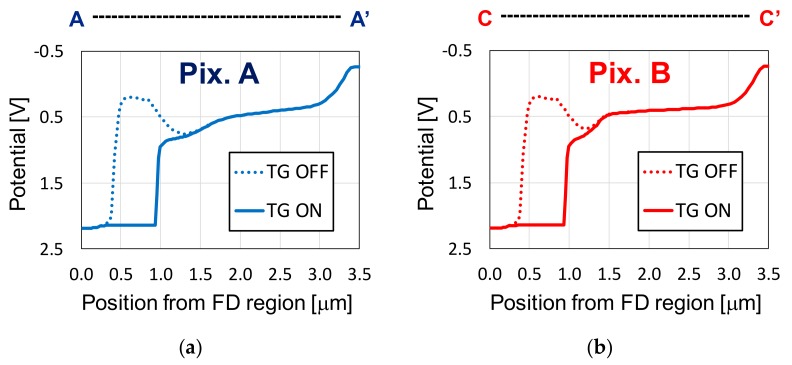
Maximum potential in the depths covered by the surface P+ and buried N layers, calculated for (**a**) Pix. A (in the transversal line A–A’), and (**b**) Pix. B (in the line C–C’). Transfer gate OFF (TG OFF) and ON (TG ON) voltages are of −0.3 and 3.3 V, respectively.

Figure 6 shows, for both Pix. A and Pix B, the simulation results of the maximum potential in the depths covered by the surface P^+^ and buried N layers, as a function of the distance from the FD region and alongside the lines of A–A’ (for Pix. A) and C–C’ (for Pix. B), respectively in Figure 6a,b. The lines A–A’ and B–B’ were previously introduced in Figure 3. The potential profile is shown for the cases of TG turned OFF and ON, where the TG voltages for OFF and ON states are −0.3 and 3.3 V, respectively.

In both pixel types, a potential gradient from the photodiode edge to the transfer gate and FD was successfully obtained, without any potential pockets inside the photodiode structure. Due to the mismatch in the internal potentials for the TG ON condition, difference in the charge transfer times between the two photodiode types are expected. This might be a concern for high speed applications, requiring more refined processes to match the profiles. Another possible mismatch between Pix. A and Pix. B is in the photodiode full well capacity (FWC). However, matching FWC in the pixel photodiodes is not necessary because, since the developed sensor features the LOFIC technology, pixel FWC is determined by the large LOFIC capacitance, instead of only the photodiodes FWC. In the developed sensor of this work, the implant conditions of Pix. A and Pix. B were optimized to achieve a photodiode FWC of 4 ke^−^ in both pixel types, and the same LOFIC capacitance was used in all pixels to avoid mismatches in the pixel FWC.

**Figure 7:**
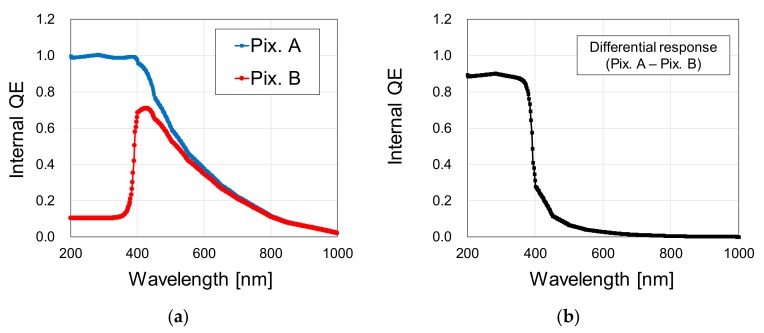
(**a**) Internal QE simulation results for the high and low UV sensitivity pixels, and (**b**) calculated differential spectral response between both pixel types.

Spectral internal QE was calculated by simulating the photocurrent generated due to a 1 × 10^−3^ W/cm^2^ light irradiation over the photodiode buried N region. The simulation was carried out in the range of 200 to 1000 nm, in 2 nm steps. Figure 7a shows the simulation results for Pix. A (blue line) and Pix. B (red line), and Figure 7b shows the differential spectral response between both pixel types, calculated by subtracting the spectral response of Pix. B from Pix. A.

The simulation results in Figure 7a show that a large difference in the UV waveband sensitivity between the high and low UV sensitivity pixels was obtained, with a very low internal QE of less than 0.1 for wavelengths longer than 800 nm in both pixel types. This confirms that the surface N^+^ layer implant is effective to reduce UV sensitivity in Pix. B, and that the Pwell implant is effective to reduce NIR light sensitivity. Figure 7b shows that, by extracting the differential spectral response, high sensitivity selectivity to the UV waveband was successfully achieved, with an internal QE of approximately 0.9 to UV light and a residual internal QE of 0.06 for 500 nm and 0.01 for 700 nm.

## 3. Chip Manufacturing and Measurement Results

**Figure 8:**
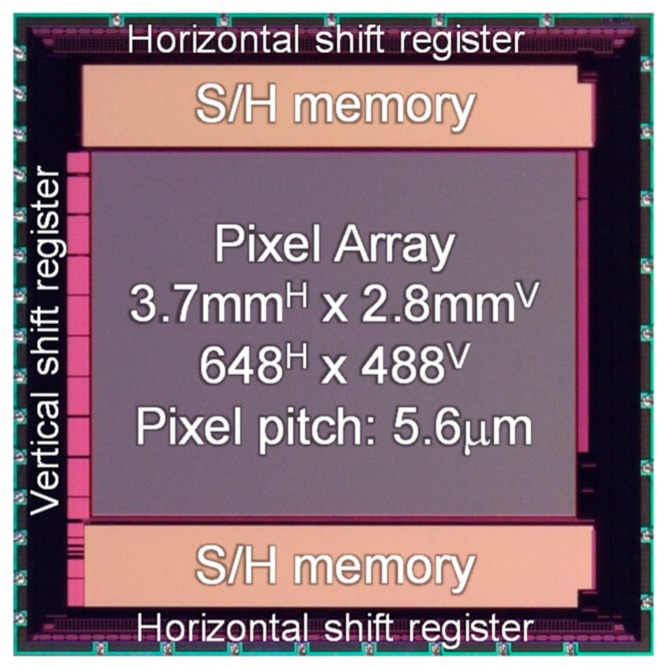
Micrograph of the developed CIS.

Figure 8 shows the manufactured chip micrograph. In this work, a 0.18 µm 1P5M CMOS with pinned PD process technology was employed. The chip power supply voltage is 3.3 V, and the chip die size is of 4.8 mm^H^ × 4.8 mm^V^. The sensor resolution is 648^H^ × 488^V^ pixels (640^H^ × 480^V^ effective), the frame rate is 30 fps and the pixel pitch is 5.6 µm. The manufactured CIS chip was measured for the photoelectric conversion curves of both high and low UV sensitivity pixels (Pix. A and Pix. B, respectively), and their respective spectral responses in terms of external QE. Sample images under UV and visible mixed light conditions are provided.

**Figure 9:**
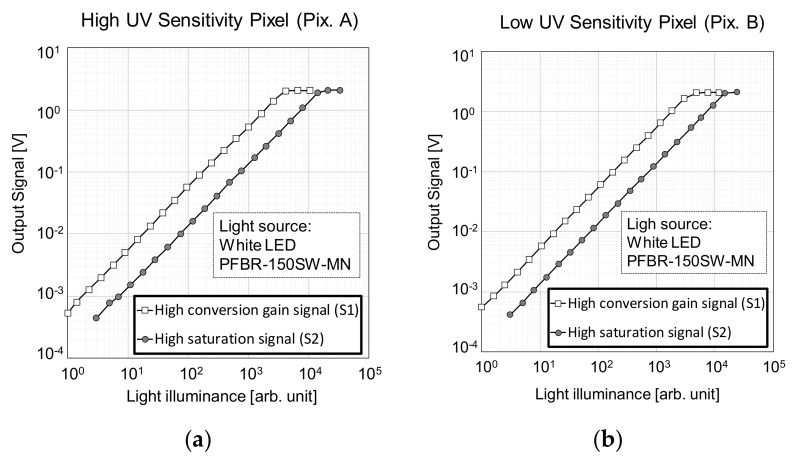
Photoelectric response characteristics for the high conversion signal S1 and the high saturation signal S2, of (**a**) the high UV sensitivity pixels, and (**b**) the low UV sensitivity pixels.

Figure 9 shows the measured photoelectric conversion curves for the high and the low UV sensitivity pixels, in Figure 9a,b, respectively. Each graph shows the curves for both the high conversion gain signal S1 and the high saturation signal S2. By combining S1 and S2 signals, a high dynamic range of 92.3 dB was obtained. The measured conversion gain was of 172 µV/e^−^ in S1 and 15.9 µV/e^−^ in S2. The saturation in S2 was of 131 ke^−^. The white LED light source PFBR-150SW-MN, manufactured by CCS Inc. (Kyoto, Japan), was used for the photoelectric conversion measurement. 

**Figure 10:**
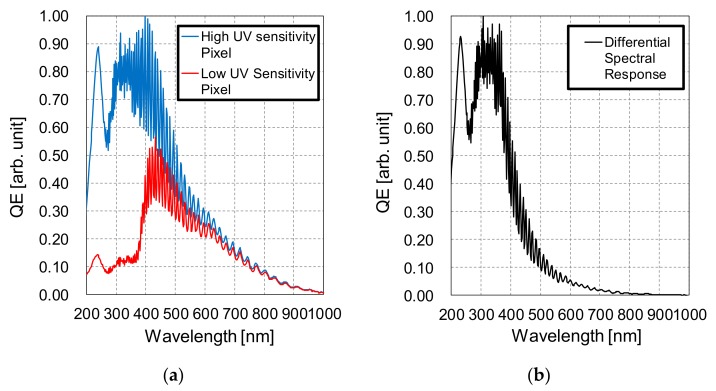
(**a**) Measured external QE for the high and low UV sensitivity pixels, respectively in the blue and red lines, and (**b**) calculated differential spectral response.

Figure 10 shows, in Figure 10a, the spectral external QE measurement results for the high UV sensitivity pixels (Pix. A) and low UV sensitivity pixels (Pix. B), and in Figure 10b, the calculated differential spectral response by subtracting the external QE of Pix. B from Pix. A. The measurement was carried out in 2 nm steps, from 200 to 1000 nm. The measurement system used consists of the EQ-99 light source, manufactured by Energetiq Technology Inc., (Wilmington, MA, USA) and the monochromator SPG-120UV, manufactured by Shimadzu Corporation (Kyoto, Japan). Calibration of the system was performed by using the photodiode S1336-18BQ, manufactured by Hamamatsu Photonics (Hamamatsu City, Japan), prior to the measurement. The results of Figure 10a show that the spectral sensitivity of Pix. A is in the range of 200 to 750 nm, and of Pix. B in the range of 390 to 750 nm. The developed sensor has silicon oxide and silicon nitride passivation layers deposited over the photodiodes, with typical thickness values of 6.6 µm and 0.2 µm, respectively. No on-chip or off-chip optical filters were employed. A high QE for the UV waveband, when compared to the visible light waveband, was obtained, as expected from the internal QE simulations shown in Figure 7. Reduced QE for visible light is due to the Pwell layer employed in both pixel types. Also, from Figure 10b, we can conclude that the resulting sensitivity after the differential spectral response extraction is in the waveband of 200 to 480 nm, therefore suitable for UV imaging. 

In the manufactured sensor, the high UV sensitivity pixel Pix. A is already an established technology. The low UV sensitivity pixel, with the surface N^+^ layer to selectively reduce UV light sensitivity, is a newly proposed structure. However, no difference in dark current between both pixel types was observed in the manufactured chip.

In order to examine the UV imaging quality by the proposed differential spectral method, the signal and noise in the differential spectral response image were also measured, for the conditions of UV light and visible light uniform illumination. The UV light illumination experiment setup consists of an UV-C (200–280 nm) germicidal lamp coupled with a diffusion plate and a band-pass filter with peak transmittance at 256 nm, full width at half maximum of 10 nm and 26% peak transmittance. The visible light illumination experiment was carried out by using the white LED light source PFBR-150SW-MN coupled with two integrating spheres, which were placed together so that the exit of one is connected to the entry port of the other.

In this measurement, 1000 frames were captured and the signal and noise of the differential image were analyzed, for each light illumination condition. Due to the UV-C germicidal lamp flicker, a frame rate of 10 fps (100 ms integration time) was used in the experiment, for both the UV-C and white LED light sources. The 648^H^ × 488^V^ pixels image captured by the developed CMOS image sensor was separated into two images, each with resolution of 344^H^ × 488^V^ pixels, by extracting alternately the pixels to obtain the Pix. A and Pix. B images separately. Then, the differential spectral response was obtained by subtracting the Pix. B image from the Pix. A image, without performing any interpolation, and the signal and noise were calculated. To achieve similar illumination conditions in both UV-C and visible light illumination experiments, the light sources intensities were adjusted to obtain approximately 5000 DN (digital number) of signal amplitude in the high saturation S2 signal of the Pix. A image. In the developed sensor, the measured saturation level of S2 was of 8115 DN. The DN signal amplitude and the number of signal electrons are in a linear relationship.

**Figure 11:**
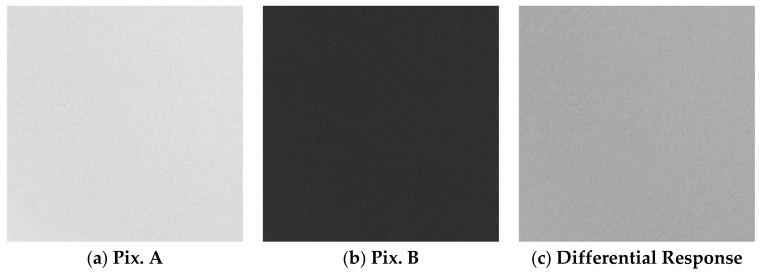
Captured images under UV-C light illumination in the conditions shown in Table 1. The image was cropped in 120^H^ × 120^V^ pixels, and no gamma or interpolation was applied.

**Figure 12:**
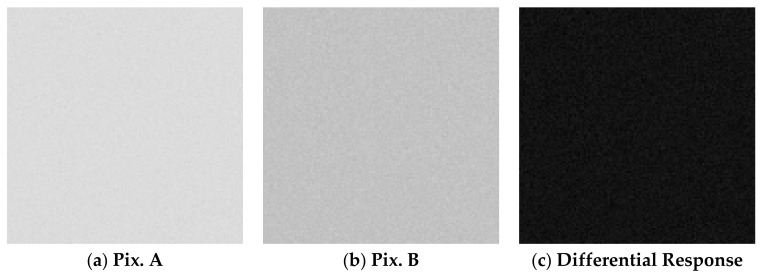
Captured images under white LED light illumination in the conditions shown in Table 1. The image was cropped in 120^H^ × 120^V^ pixels, and no gamma or interpolation was applied.

The measurement results of the Pix. A output signal amplitude and of the differential response image signal amplitude and standard deviation (noise) are summarized in Table 1. The presented data is the average value of all the effective 340^H^ × 480^V^ pixels. The signal amplitude in the Pix. A image was of 5317 DN for the UV-C light source and 5111 DN for the visible light source. The white LED light source illuminance was of 24.8 LUX. The captured images under the UV-C and white LED light sources are shown in detail in Figure 11 and Figure 12, respectively. Both figures show a 120^H^ × 120^V^ pixels crop of the same region. No interpolation or gamma processing was applied.

From the results, when using a UV-C light source, the signal amplitude in the differential spectral response image is lower than the UV signal in the Pix. A image. This is because the residual UV sensitivity in Pix. B partially reduces the UV signal after extraction. The measurement under visible light (white LED) shows that, after the differential spectral response extraction, the visible light signal amplitude is largely reduced, but the noise in the image is increased when compared to the condition of UV-C light source illumination. This is because, when extracting the differential spectral response, the noise of Pix. A and Pix. B are added in the subtraction process, while the signal is cancelled out. This shows that the background visible light deteriorates the differential response image quality, because it is detected by both Pix. A and Pix. B, therefore producing a higher photon shot noise without adding to the signal. This effect should be considered when performing UV imaging of weak UV light under strong background visible light conditions.

**Figure 13:**
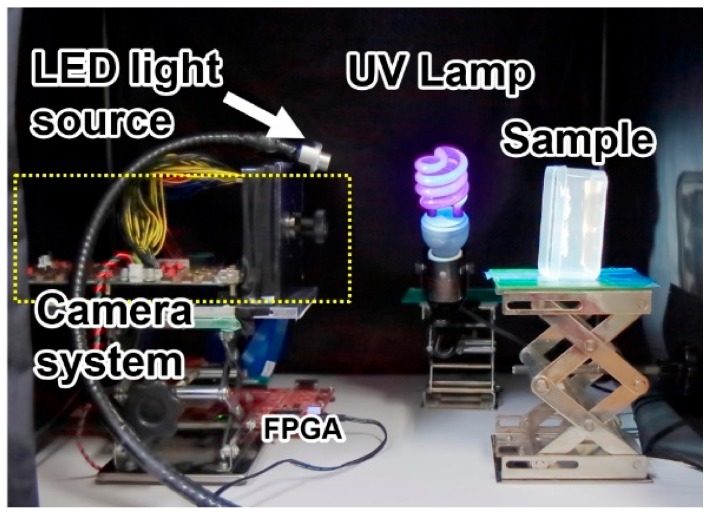
Setup employed to capture sample images using the developed CIS.

As a simple example of UV and visible light imaging, the setup shown in Figure 13 was used to capture UV and visible light images of white cabbage butterflies simultaneously, using the manufactured CIS. In this setup, the butterflies were illuminated by the 20 W UV lamp EFD23-SSBK, manufactured by Jefcom (Osaka, Japan), and the white LED light source PFBR-150SW-MN, manufactured by CCS Inc. (Kyoto, Japan). The images were captured using the high UV transmittance lens UV1228CM, with 6 mm focal length and maximum aperture of F3.5, manufactured by UNIVERSE OPTICAL INDUSTRIES Co. ltd., (Tatsuno, Nagano, Japan). No optical filters were employed in this experiment, and the images were captured simultaneously in a single exposure.

**Figure 14:**
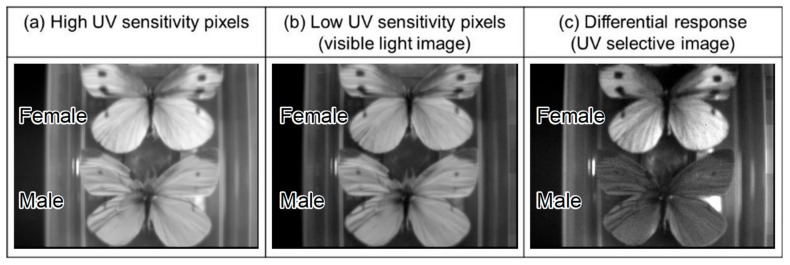
Female (top) and male (bottom) cabbage white butterflies’ images captured by the developed CMOS image sensor. Here, (**a**) shows the output from high UV sensitivity pixels, (**b**) shows visible light image captured from the low UV sensitivity pixels, and (**c**) shows the UV selective image obtained by the differential response extraction. The images were captured in a single exposure simultaneously, and without using optical filters.

The captured images are shown in Figure 14a, for the high UV sensitivity pixels, Figure 14b for the low UV sensitivity pixels, therefore showing the captured visible light image, and Figure 14c for the calculated differential response, therefore showing the UV selective image. In these images, a simple interpolation consisting of averaging the adjacent pixels was carried out, and a gamma of 0.45 was applied. Both male and female white cabbage butterflies reflect visible light, but only the male absorbs UV light, a feature that was successfully observed in the captured images. Therefore, we successfully accomplished UV-selective and visible light imaging simultaneously, without employing optical filters neither time shared illumination techniques. The developed sensor can be used in applications that require UV imaging under variable background visible light, and also applications requiring simultaneous UV and visible imaging with a simple optical system. The performance summary of the manufactured sensor is summarized in Table 2.

## 4. Conclusions

In this work, we developed a 648^H^ × 488^V^ pixels (640^H^ × 480^V^ effective) CIS with high and low UV light sensitivity pixels arranged in a checker pattern. The pixels were designed to match for sensitivity outside the UV waveband, and the sensor is capable of filter-less UV imaging by extracting the differential response between both pixel types. Simultaneously, the visible light image is obtained from the low UV sensitivity pixels in a single exposure. The developed sensor has shown a high conversion gain of 172 µV/e^−^, a high saturation of 131 ke^−^, and a wide dynamic range of 92.3 dB by employing the LOFIC technology. The obtained spectral sensitivity ranges were of 200–750 nm in the high UV sensitivity pixels, 390–750 nm in the low UV sensitivity pixels and 200–480 nm in the differential spectral response image, showing that high selectivity to the UV waveband was obtained from the differential spectral response extraction. The developed CIS requires only a single additional photomask and a few additional ion implantation steps to be manufactured, and it is promising for various sensing applications that require UV light imaging under strong or variable background visible light, such as flame detection, food freshness inspection and so on.

## Figures and Tables

**Table 1:** Differential response characteristics under UV-C and visible light source.

Light Source	Pix. A Image Signal Amplitude	Differential Response Image	
		Signal Amplitude	Noise
UV-C	5317 DN	4102 DN	31.12 DN
White LED	5111 DN	588 DN	36.56 DN

**Table 2:** Performance summary of the developed CIS.

Process Technology		0.18 µm 1P5M CMOS with Pinned PD	
Power Supply Voltage		3.3 V	
Die Size		4.8 mm^H^ × 4.8 mm^V^	
Pixel Size		5.6 µm^H^ × 5.6 µm^V^	
Number of Pixels	Total	648^H^ × 488^V^	
	Effective	640^H^ × 480^V^	
Aperture Ratio		36%	
Frame Rate		30 fps	
Conversion Gain		172 µV/e^−^ (S1 signal)	
Full Well Capacity		131 ke^−^ (S2 signal)	
Dynamic Range		92.3 dB	
Spectral Sensitivity Range		High UV Sensitivity Pixel	200–750 nm
		High UV Sensitivity Pixel	390–750 nm
		Differential Response	200–480 nm
